# The prognostic value of the tumour marker Cyfra 21-1 in carcinoma of head and neck and its role in early detection of recurrent disease

**DOI:** 10.1054/bjoc.2000.1502

**Published:** 2000-12

**Authors:** I Doweck, M Barak, N Uri, E Greenberg

**Affiliations:** Department of Otolaryngology, Head and Neck Surgery, Carmel Medical Center, 7 Michal St., Haifa, 34362; Haifa and Western Galilee Central Laboratory, Haifa, Israel

**Keywords:** head and neck cancer, tumour marker, Cyfra 21-1, prognostic factor, recurrent cancer

## Abstract

This study examines a new tumour marker, Cyfra 21-1, as a prognostic marker in predicting the survival of H&N cancer patients, and its correlation with clinical outcome during prolonged follow up of these patients. The study included 67 patients with primary detection of carcinoma of H&N. The survival of these patients was evaluated in correlation with the disease stage and Cyfra 21-1 levels at initial diagnosis. 38 patients were followed clinically and with serial assays for at least 12 months, or until recurrence was diagnosed. Cyfra 21-1 levels were determined periodically, using an Elisa kit. Patients with Cyfra 21-1 < 1.5 ng ml^–1^had a higher survival rate compared to patients with Cyfra 21-1 ≥ 1.5 ng ml^–1^(63% vs. 20%, respectively). The risk ratio of Ln(Cyfra 21-1) is 1.62 (*P* = 0.028). In a Cox regression model that included the disease stage and Ln(Cyfra 21-1), Ln(Cyfra 21-1) was preferred as the main parameter for predicting patients survival. In 83% of the 12 patients with recurrent or residual disease, Cyfra 21-1 was elevated before or during clinical detection of the recurrence. Cyfra 21-1 was found to be a prognostic marker for carcinoma of H&N, unrelated to the stage of the disease. Elevated levels of Cyfra 21-1 without clinical evidence of disease can be attributed to the marker's mean lead-time as compared to the clinical appearance of the disease. © 2000 Cancer Research Campaign http://www.bjcancer.com


*British Journal of Cancer* (2000) **83**(12), 1696–1701
© 2000 Cancer Research Campaign
doi: 10.1054/ bjoc.2000.1502, available online at http://www.idealibrary.com on IDEAL®

http://www.bjcancer.com

### The prognostic value of the tumour marker Cyfra 21-1 in carcinoma of head and neck and its role in early detection of recurrent disease

**I Doweck,[1] M Barak,[2] N Uri[1] and E Greenberg[1]**

[1]Department of Otolaryngology, Head and Neck Surgery, Carmel Medical Center, 7 Michal St., Haifa 34362; [2]Haifa and Western Galilee Central Laboratory, Haifa, Israel

**Summary** This study examines a new tumour marker, Cyfra 21-1, as a prognostic marker in predicting the survival of H&N cancer patients, and its correlation with clinical outcome during prolonged follow up of these patients. The study included 67 patients with primary detection of carcinoma of H&N. The survival of these patients was evaluated in correlation with the disease stage and Cyfra 21-1 levels at initial diagnosis. 38 patients were followed clinically and with serial assays for at least 12 months, or until recurrence was diagnosed. Cyfra 21-1 levels were determined periodically, using an Elisa kit. Patients with Cyfra 21-1 $< 1.5$ ng ml$^{-1}$ had a higher survival rate compared to patients with Cyfra 21-1 $\geq 1.5$ ng ml$^{-1}$ (63% vs. 20%, respectively). The risk ratio of Ln(Cyfra 21-1) is 1.62 ($P = 0.028$). In a Cox regression model that included the disease stage and Ln(Cyfra 21-1), Ln(Cyfra 21-1) was preferred as the main parameter for predicting patients survival. In 83% of the 12 patients with recurrent or residual disease, Cyfra 21-1 was elevated before or during clinical detection of the recurrence. Cyfra 21-1 was found to be a prognostic marker for carcinoma of H&N, unrelated to the stage of the disease. Elevated levels of Cyfra 21-1 without clinical evidence of disease can be attributed to the marker's mean lead-time as compared to the clinical appearance of the disease. © 2000 Cancer Research Campaign http://www.bjcancer.com

**Keywords**: head and neck cancer; tumour marker; Cyfra 21-1; prognostic factor; recurrent cancer

Many efforts have been dedicated to identify an adequate tumour marker in the serum of patients with squamous cell carcinoma of head and neck (HNSCC), which would allow for simple non-invasive follow up of these patients. Unfortunately, there is no known reliable tumour marker for HNSCC. Detection of recurrent disease among these patients is entirely dependent on clinical criteria: symptoms, physical examination and radiological studies such as CT scan and MRI. However, the ability of the clinician to detect recurrent cancer in head and neck cancer patients after radiotherapy or surgery to the head and neck at an early stage is severely limited. Recurrent disease is often deep seated and obscured by soft tissue and bone that often is altered by radiation and surgical damage.

Although several serum tumour markers were tested in HNSCC, none have proven to be clinically beneficial. Early studies reported very low sensitivity of various markers like CEA (carcinoembryonic antigen) (Silverman et al, 1976; Walther et al, 1990), Lipid associated sialic acid (Katopodis et al, 1982; Ropka et al, 1991) and SCC-marker (TA-4) (Calsen et al, 1990; Daver et al, 1990; Fichbach et al, 1990; Walther et al, 1990; Yoshimura et al, 1990; Ropka et al, 1991) in head and neck cancer patients. Cyfra 21-1 was reported as a new marker for non-small cell carcinoma of the lung (Pujol et al, 1993; Stieber et al, 1993; Rastel et al, 1994). For SCC of the lung, the sensitivity was 60% and specificity was 95%. In non-small cell lung cancer, especially squamous cell carcinoma, Cyfra 21-1 was found to be a prognostic indicator. Patients with a serum level of Cyfra 21-1 above an arbitrary defined cut-off had a significantly shorter overall survival than those with Cyfra 21-1 below the cut-off (Pujol et al, 1993).

In a preliminary study with small group of patients with HNSCC, Cyfra 21-1 levels were found to be higher in patients with squamous cell carcinoma of head and neck than in patients with benign tumours of head and neck, in primary detection of the disease. The sensitivity and specificity of the marker were 60% and 94%, respectively. Positive and negative predictive values were 75% and 89% (Doweck et al, 1995).

Despite the high levels of Cyfra 21-1 at the time of the initial diagnosis of head and neck cancer, its prognostic value in a long-term follow-up has never been proven. More than that, the sensitivity and the mean lead-time of the marker in detecting recurrent cancer of head and neck, were not previously reported.

Cyfra 21-1 is a cytokeratin 19 fragment that is recognized by the two monoclonal antibodies BM 19-21 and KS 19-1, which were obtained by immunization of mouse with MCF-7 cells (Bodenmuller et al, 1992). Cytokeratin 19 is an acid-type cytokeratin, with a molecular weight of 40 000 d, and it is released into the serum in the form of soluble fragments. It is a cytoplasmatic protein, one of the cytokeratins which form the intermediate filament cytoskeleton within epithelial cells (Moll et al, 1982; Osborn and Weber, 1982). The cytokeratins appear to be distributed in the various epithelia, according to the cell differentiation (Hoefler and Derk, 1984). During the malignant transformation, the epithelial cells appear to contain the same cytokeratins as do normal cells. The simple epithelium contains cytokeratins 8, 9, 18 and 19 (Moll et al, 1982).

*Received 8 May 2000*
*Revised 16 August 2000*
*Accepted 17 August 2000*

*Correspondence to*: I Doweck

In the present study, we correlated the expression of the serum marker Cyfra 21-1 at the initial diagnosis with the survival rate of head and neck cancer patients, and we evaluated the detection of recurrence of cancer by repeated blood samples during long term follow-up. The present study also determined the mean lead-time between the elevation of the marker and the clinical detection of recurrence among disease-free patients undergoing serial assays during follow-up.

### PATIENTS AND METHODS

### Patients

The study was prospective, and included 67 patients with cancer of head and neck, untreated previously. The histological diagnosis revealed squamous cell carcinoma in 57 patients and adenocarcinoma in 5 patients. (Other histological diagnoses included: mucoepidermoid carcinoma, adenoid cystic carcinoma, and undifferentiated carcinoma.) The sites of origin of the tumors are: larynx – 34 patients, carcinoma with unknown primary – 9 patients, nasopharynx – 6 patients, parotid gland – 6 patients, nose and paranasal sinuses – 4 patients, oropharynx – 3 patients, ear – 3 patients and oral cavity – 2 patients.

The stage of the disease was determined using the TNM classification according to the American Joint Committee of Cancer's (AJCC) staging system. The T staging of the patients was: T1 – 16 patients, T2 – 13 patients, T3 – 18 patients, T4 – 11 patients, Tx (primary tumour cannot be assessed) – 9 patients. The N staging was: N0 – 37 patients, N1 – 4 patients, N2 – 21 patients, N3 – 5 patients. The stage of the disease: stage 1 – 15 patients, stage II – 9 patients, stage III – 9 patients, stage IV – 34 patients.

Blood samples for Cyfra 21-1 levels were collected after informed consent was obtained, and before treatment was started.

### Cyfra 21-1

Cyfra 21-1 was evaluated using an ELISA kit (Elsa Cyfra 21-1 IRMA Kit, CIS Biointernational, Gif-SurYvette, France). The mean, standard deviation, median, and range of Cyfra 21-1 level at the various T, N, and stage classifications are shown in Table 1.

### Survival rates

The correlations between Cyfra 21-1 levels at initial diagnosis and the survival rates of the patients were evaluated using the following parameters:

1. Patient status – alive or dead – obtained from official government records.
2. Duration of follow up (from the time of diagnosis).
3. Cyfra 21-1 levels at the time of initial diagnosis. A logarithmic transformation of the parameter Cyfra 21-1, Ln(Cyfra) was used for further analysis.
4. The stage of the disease at initial diagnosis.

### Follow-up and detection of recurrent disease

For the evaluation of Cyfra 21-1 as an early indicator of recurrent disease, the patients were followed up throughout the treatment period regardless of the treatment category. Follow-up was carried out every month during the first year after diagnosis, and every 3 months in the following years. Follow-up included history, physical examination and blood samples of 5 ml each for determination of Cyfra 21-1 levels. Clinical suspicion of recurrence was further investigated by diagnostic tools such as CT scan or MRI, and biopsy was done to confirm the diagnosis. If significant increase of Cyfra 21-1 levels (>25% from the previous marker level) was detected without clinical evidence of recurrent disease, blood sample was repeated. If the elevation in the marker level was constant, patients were further investigated by non-invasive diagnostic tools (radiography, CT, etc.).

Only patients with follow-up of more than 12 months were included in this analysis. These included 38 patients. (Of the initial group of 67 patients, 11 patients suffered from progressive disease and they died of the disease within the first year of follow up, 4 patients were followed without using this marker, and 14 patients were followed-up for less than 12 months until the end of the study.)

### Statistical analysis

The survival rates were estimated using the Kaplan–Meier survival table, both in relation to Cyfra 21-1 levels and to the stage of the disease, and the differences between the curves in each survival

**Table 1** Cyfra 21-1 levels (mean, standard deviation [SD], median, and range [ng/ml]) in correlation to the different staging subgroups: T1–4, Tx – primary tumour cannot be assessed, N0–3, and Stage I–IV

| Cyfra 21-1 (ng ml$^{-1}$) | T1 (*n* = 16) | T2 (*n* = 13) | T3 (*n* = 18) | T4 (*n* = 11) | Tx (*n* = 9) |
|---|---|---|---|---|---|
| Mean | 1.34 | 2.31 | 2.24 | 2.91 | 4.23 |
| SD | 0.68 | 4.06 | 1.81 | 1.56 | 6.65 |
| Median | 1.20 | 1.05 | 1.70 | 2.20 | 1.50 |
| Range | 0.43–2.8 | 0.33–15.7 | 0.2–6.6 | 0.92–5.9 | 0.8–21 |
| | **N0 (*n* = 37)** | **N1 (*n* = 4)** | **N2 (*n* = 21)** | **N3 (*n* = 5)** | |
| Mean | 1.87 | 1.60 | 2.52 | 6.70 | |
| SD | 2.55 | 0.48 | 1.81 | 8.44 | |
| Median | 1.25 | 1.75 | 1.85 | 2.40 | |
| Range | 0.2–15.7 | 0.92–2 | 0.72–6.6 | 0.9–21 | |
| | **Stage I (*n* = 15)** | **Stage II (*n* = 9)** | **Stage III (*n* = 9)** | **Stage IV (*n* = 34)** | |
| Mean | 1.24 | 2.73 | 1.73 | 3.03 | |
| SD | 0.58 | 4.90 | 1.12 | 3.67 | |
| Median | 1.15 | 1.05 | 1.70 | 1.88 | |
| Range | 0.43–2.5 | 0.33–15.7 | 0.2–4.3 | 0.45–21 | |

*n* – number of patients in each subgroup.

© 2000 Cancer Research Campaign table were obtained using the Log Rank test. A Cox regression model was used once to evaluate the risk ratio between low and high levels of Cyfra 21-1, and a second time to include both the stage of the disease and the Ln(Cyfra 21-1) variables. The definition of low and high Cyfra 21-1 levels was according to cut-off, 1.5 ng $ml^{-1}$, for which the sensitivity and specificity are 55% and 96%, respectively; positive predictive value and negative predictive values are 79% and 89%, respectively. Low Cyfra 21-1 was Cyfra 21-1 <1.5 ng $ml^{-1}$, while high level was Cyfra 21-1 ≥ 1.5 ng $ml^{-1}$.

The statistical analysis was performed using SPSS software for Windows.

### RESULTS

The study included 67 patients, 56 male and 11 female, whose mean age was 64 ± 13 years (median 64, range 37–90 years). The patients were followed-up for a mean time of 18 ± 11.7 months, (median 15; range 1–44 months). Mean follow up duration for surviving patients (38 patients) was 21.6 ± 12.5 months (median 20.5; range 1–44 months), and 13.6 ± 8.9 months (median 12; range 2–37 months) for 29 terminally ill patients.

32 patients had low levels of Cyfra 21-1 at diagnosis (< 1.5 ng $ml^{-1}$), 23 of them (72%) are alive and 9 (28%) died. 35 patients had high levels of Cyfra 21-1 (≥1.5 ng $ml^{-1}$) at diagnosis; of these, 15 are alive (43%) and 20 died (57%).

### Survival rates

Figure 1 presents the survival rate of all the patients included in the study. Using the Kaplan–Meier survival table, Figure 2 compares cumulative survival rates of patients with low and high Cyfra 21-1 levels. According to Figures 1 and 2, the survival rate of all patients is 37% for 44 months. Patients with Cyfra 21-1 levels below 1.5 ng $ml^{-1}$ had a survival rate of 63% for 44 months, in comparison with a 20% survival rate for those with Cyfra 21-1 levels exceeding 1.5 ng $ml^{-1}$. These differences are borderline significant: P = 0.055 in the Log Rank test.

A Cox regression model for evaluating the risk ratio between low and high levels of Cyfra 21-1 using Ln(Cyfra 21-1) found the Odd's ratio of Ln(Cyfra 21-1) to be 1.62, which is significant ($P$ = 0.028).

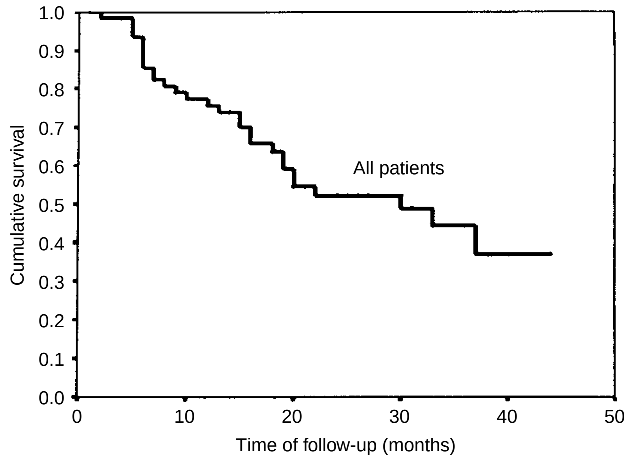

**Figure 1** Cumulative survival rates of all patients

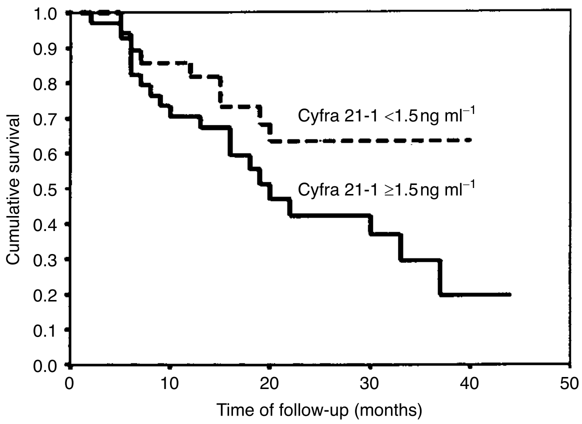

**Figure 2** Survival rates of patients with high (≥ 1.5 ng $ml^{-1}$) and low (< 1.5 ng $ml^{-1}$) Cyfra 21-1 levels

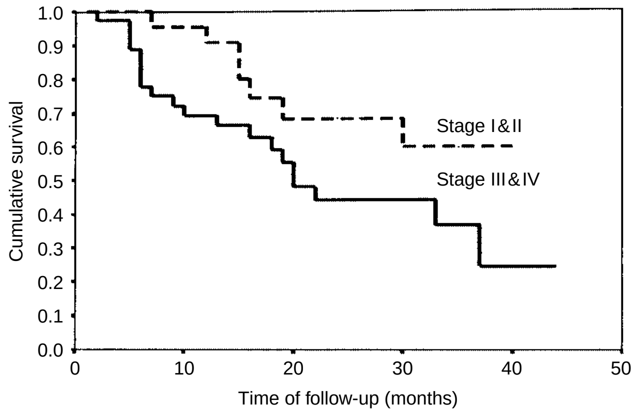

**Figure 3** Cumulative survival rates of patients with disease at stages I and II compared to patients at stages III and IV

The survival rate according to the stage of the disease is shown in Figure 3. According to the Kaplan–Meier survival table, patients in stages I and II had a survival rate of 60% for 44 months, in comparison with a 24% survival rate over 44 months in subjects in stages III and IV. The differences are borderline significant: $P$ = 0.06 in the Log Rank test.

A Cox regression was performed in which the stage of the disease and Ln(Cyfra 21-1) levels were included. This model found that both the stage and the Ln(Cyfra 21-1) are related to survival. However, the Cox Regression model prefers Ln(Cyfra 21-1) as the main parameter for predicting the survival of patients with carcinoma of H&N, with $P$ = 0.047.

### Follow up and detection of recurrent disease

38 patients were followed clinically and biochemically for over 12 months, or until recurrence was diagnosed. During the extended follow up, 14 patients died. The mean time of follow up of all 38 patients was 23.4 ± 10.2 months; for surviving patients 26.6 ± 10 months; for terminal patients 17.9 ± 8.2 months.

12 (31.6%) of the 38 patients were diagnosed with recurrent or residual disease. 26 (68.4%) patients showed no evidence of disease during the follow-up period. 10 (83%) of the 12 patients

 *© 2000 Cancer Research Campaign*

**Table 2** The clinical data of the patients with recurrent disease including: the site of origin of the disease, histology, TNM, stage, Cyfra 21-1 level at the initial diagnosis, after treatment and at recurrence and the mean lead time between the elevation of the marker level and the clinical diagnosis of the recurrent disease.

| Patient | Site | Histology | TNM | Stage | Cyfra 21-1 at diagnosis (ng ml$^{-1}$) | Treatment | Cyfra 21-1 after treatment (ng ml$^{-1}$) | Cyfra 21-1 at recurrence (ng ml$^{-1}$) | Lead time (months) |
|---|---|---|---|---|---|---|---|---|---|
| A.J. | Glottis | SCC, wd | T3N2M0 | IV | 1.9 | Surgery + RT | 1.0 | 1.6 | 0 |
| M.Z. | Supraglottic | SCC, md | T3N2M0 | IV | 4.0 | Surgery | 0.9 | 1.7 | 6 |
| S.J. | Nasopharynx | SCC, nk | T4N2M0 | IV | 3.2 | CHT + RT | 0.3 | 1.55 | 6 |
| B.A. | Base of tongue | SCC, md | T2N0M0 | II | 2.25 | RT | 1.6 | 2.05 | 10 |
| K.Z. | Glottis | SCC, wd | T1N0M0 | I | 2.1 | RT | 2.2 | 2.2 | 2 |
| P.G. | Glottis | SCC, wd | T1N0M0 | I | 0.92 | RT | 1.65 | 1.95 | 0 |
| V.R. | Glottis | SCC, wd | T1N0M0 | I | 0.86 | RT | 1.7 | 1.7 | 0 |
| F.S. | Parotid gland | SCC, md | T3N2M0 | IV | 6.0 | Surgery | 1.5 | 3.9 | 6 |
| A.H. | Glottis | SCC, md | T1bN0M0 | I | 2.5 | Surgery | 1.8 | 2.2 | 1 |
| K.E. | Parotid gland | Adenoca. | T3N0M0 | III | 2.2 | Surgery + RT | 5.8 | 24 | 8 |
| Z.M. | Ear | SCC, wd | T4N0M0 | IV | 1.3 | CHT +RT | 1.0 | 1.0 | no elevation |
| C.F. | Glottis | SCC, wd | T4N1M0 | IV | 0.92 | Surgery +RT | 0.61 | 1.06 | no elevation |

wd – well differentiated, md – moderately differentiated, nk – non-keratinizing, adenoca. – adenocarcinoma, SCC – squamous cell carcinoma, RT – radiotherapy, CHT – chemotherapy.

with recurrent disease showed significant elevation of Cyfra 21-1 levels before or concomitantly with diagnosis of recurrent or residual disease. Of the 12 patients with recurrent disease, 50% showed elevated Cyfra 21-1 levels before the clinical detection of the disease, 33% of the patients had elevated marker levels concomitantly with the clinical detection. The mean time between the elevation of the marker and the diagnosis of clinical recurrence (mean lead-time) was 4.1 ± 3.4 months. In two patients (17%) with clinical evidence of recurrent disease, Cyfra 21-1 levels were not elevated at the time of diagnosis of the recurrence. The two patients had nothing in common except the low initial levels of Cyfra 21-1. Table 2 summarizes the clinical data, Cyfra 21-1 levels, and the lead-time between the elevation in the marker level and the diagnosis of recurrent disease in these patients.

26 patients showed no evidence of disease for at least 12 months of follow up. In 20 of them (77%), there was no significant elevation in marker levels during follow up. In radiated patients there was constant elevation in marker levels, both during treatment and up to 2–3 months after treatment was completed. 4 patients (15%) exhibited no evidence of disease, but a fluctuation in the marker levels was noted. None of them had a constant elevation. Two patients (15%) who exhibited no clinical evidence of the disease had a significant elevation of Cyfra 21-1 and died shortly thereafter.

### DISCUSSION

Elevated levels of Cyfra 21-1 in previously untreated patients with head and neck cancer were reported in few studies. Our preliminary data showed high levels of Cyfra 21-1 in HNSCC (Doweck et al, 1995). The sensitivity of the marker in nasopharyngeal carcinoma was found to be 58.3% (Yen et al, 1998). Ogawa et al reported 41.7% sensitivity of Cyfra 21-1 in patients with HNSCC (Ogawa et al, 1999). However, the prognostic value of Cyfra 21-1 levels in a long-term follow up of these patients was not reported before.

In the present study we found that patients with Cyfra 21-1 levels below 1.5 ng ml$^{-1}$ had a higher survival rate, 63%, compared to 20% for patients whose Cyfra 21-1 levels were above 1.5 ng ml$^{-1}$. The Odd's ratio is 1.62 for the logarithmic transformation of Cyfra 21-1, Ln (Cyfra 21-1). Elevation of 1 Ln (Cyfra 21-1), which is equivalent to elevation of Cyfra 21-1 levels by ~2.7 fold, increases the death risk by 62%.

The prognostic value of Cyfra 21-1 was reported previously in non-small cell lung cancer, especially in SCC, by Pujol et al (Pujol et al, 1993), who found that patients with a serum Cyfra 21-1 level above cut-off had a significantly shorter overall survival than those with a normal serum level. Similar findings were reported by Niklinski et al (Niklinski et al, 1995), and by Ebert and colleagues (Ebert et al, 1995). As shown in this study, Cyfra 21-1 is the serum tumour marker with the most accurate prognostic value for patients of carcinoma of head and neck.

Staging of the disease by the TNM classification according to the AJCC is accepted worldwide. In addition to providing important clinical guidelines, the TNM classification has a well-established prognostic value. In the present study, the TNM classification was again found to be a prognostic factor in cancer of the head and neck. Interestingly, Cyfra 21-1 level (in a logarithmic transformation) was found to be the main parameter for predicting survival in this group of patients. This finding was unexpected; however, the correlation between the serum Cyfra 21-1 level and the survival rate was expected. A previous study found that Cyfra 21-1 levels were indeed correlated to the extent of the disease, as expressed by the T and N classifications in squamous cell carcinoma of head and neck (Doweck et al, 1995). However, in our study, the prognostic value of Cyfra 21-1 is independent of the stage of the disease. Our findings are in agreement with those of Pujol et al (Pujol et al, 1993, 1996), who report that the prognostic significance obtained by Cyfra 21-1 in squamous cell lung cancer is independent and adds to the information obtained from other classic prognostic factors, such as extent of the disease, weight loss, performance status, etc. Niklinski and colleagues report that Cyfra 21-1 may be an independent prognostic parameter of survival and tumour relapse in squamous cell lung cancer (Niklinski et al, 1996).

Cyfra 21-1, as reported before, is a soluble fragment of cytokeratin 19. The assumption is that Cyfra 21-1 is released into the bloodstream during cell death, and therefore its level correlates very well with the tumour mass, or more specifically with the necrosis in the tumour, which is a function of the tumour mass.

© 2000 Cancer Research Campaign 

The finding that Cyfra 21-1 levels may be an independent marker and the preferred prognostic factor in head and neck cancer may indicate that this marker reflects tumour mass more accurately than it does the stage of the disease as expressed by the TNM. This finding may also have a therapeutic implication, as the tumour mass is one of the main parameters in deciding a therapeutic regimen. Since there is no other study on the prognostic value of Cyfra 21-1 in head and neck cancer, this finding should be carefully examined.

The detection of circulating markers has the potential to serve as a simple minimally invasive and inexpensive technique for monitoring patients for recurrent cancer. In the case of the head and neck, there is no circulating marker currently available for this purpose. In the present study, the clinical follow-up of the patients was accompanied by periodic monitoring of Cyfra 21-1 levels. 83% of the patients with recurrent disease showed elevated Cyfra 21-1 levels before or concomitantly with clinical detection of the recurrent disease. 77% of patients with no evidence of disease also had no elevation in the marker level. Previous studies show that Cyfra 21-1 levels drop to below cut-off levels 24 hours after successful operation (Ebert et al, 1994; Van der Gaast et al, 1994; Doweck et al, 1995). According to our findings, re-elevation of the marker had a positive predictive value of 75% (10 cases of recurrence, 2 deaths, 4 NED). These findings are in agreement with the results reported by Niklinski et al in squamous cell lung cancer (Niklinski et al, 1996). Similar findings in squamous cell lung cancer were also reported by Van der Gaast et al (Van der Gaast et al, 1994). Currently no other circulating tumour marker is able to provide similar sensitivity for detecting recurrent cancer of the head and neck.

It is important to emphasize that according to this study, the marker had a mean lead-time of 4.1 months, in which the increased marker levels predicted the clinical detection of the recurrent disease. Two patients with recurrent disease did not present with elevated marker levels. As reported, they had nothing in common except low initial levels of Cyfra 21-1. The absence of elevated Cyfra 21-1 levels, both at diagnosis and at recurrence, can be explained by a low tumour mass or by the inability of these tumour cells to secrete cytokeratin 19 fragments.

Elevated levels of Cyfra 21-1 were found in patients who were treated with radiotherapy during the treatment and up to 2–3 months after completing the treatment. The constant high levels of Cyfra 21-1 during radiotherapy may indicate continuous cells damage and tumour necrosis during radiation. Since radiation affects tumour two months after it is ended, one might expect high levels of Cyfra 21-1 during this period due to continuous cells damage.

As far as we know, in the field of head and neck, there is no other tumour marker that can provide a similar correlation between marker levels and the progression of the disease.

According to our findings, by means of a simple, noninvasive blood test, Cyfra 21-1 levels provide the head and neck oncologist with a prognostic tool and an additional monitoring system for early recognition of the progression of the disease. However, since the study included a relatively small group of patients, further investigations encompassing larger patient groups and in a particular head and neck cancer sub-site are necessary before the marker can be used routinely in patients with carcinoma of the head and neck.

### REFERENCES

Bodenmuller H, Banauch D, Ofenloch B, Jaworek D and Dessauer A (1992) Cancer of the breast – state and trends in diagnosis and therapy. In: Klapdor R (ed) Technical evaluation of a new automated tumour marker assay: the enzymun-test Cyfra 21-1. Tumour Associated Antigens, Oncogenes, Receptors, Cytokines in Tumour Diagnosis and Therapy at the Beginning of the Nineties. W Zuckschwerdt Verlag Munchen, Bern, Wien, pp 137–138

Bonfrer JMG, Gaarenstroom KN, Kenter GG, Korse CM, Hart AAM, Gallee MPW, Helmerhorst TJM and Kenemans P (1994) Prognostic significance of serum fragments of cytokeratin 19 measured by Cyfra 21-1 in cervical cancer. *Gynecologic Oncology* **55**: 371–375

Calsen B, Pere P, Senekowitsch R and Menz E (1990) SCC as a tumor marker in the initial diagnosis of carcinoma of the head and neck region. *Laryngorhinootologie* **65**: 275–280

Daver A, Dalifard I, Pns-Anicet D, Krebs BP, Gosselin P, Cazin JL, Ricolleau G, Gaillard G, Gachon F and Goussard J et al (1990) Diagnostic value of SCC-TA4 determination in four localization of epidermoid cancer: an experience of the FNCLCC subgroup of radio-analysis. *Bull Cancer* (Paris) **77**: 781–792

Doweck I, Barak M, Greenberg E, Uri N, Kellner J, Lurie M and Gruener N (1995) Cyfra 21-1 a new potential tumor marker for squamous cell carcinoma of head and neck. *Arch Otolaryngol Head Neck Surg* **121**: 177–181

Ebert W, Dienemann H, Fateh-Moghadam A, Scheulen M, Konietzko N, Schleich T and Bombardieri E (1994) Cytokeratin 19 fragment Cyfra 21-1 compared to carcinoembrionic antigen, squamous cell carcinoma antigen and neuron specific enolase in lung cancer. Result of an international multicentric study. *Eur J Clin Chem Clin Biochem* **32**: 189–199

Ebert W, Bodenmuller H and Holzel W (1995) Cyfra 21-1 – clinical application and analytical requirements. *Scan J Clin Lab Invest* **55** Suppl 221: 72–80

Fichbach N, Meyer T and Barthel K (1990) Squamous cell carcinoma antigen in the diagnosis and treatment follow-up of oral and facial squamous cell carcinoma. *Cancer* **65**: 1321–1324

Hoefler H and Denk H (1984) Immunohistochemical demonstration of cytokeratin in gastrointestinal carcinoids and their probable precursors cells. *Virchows Arch. B. Cell Pathol* **403**: 235–240

Katopodis N, Hishaut Y, Geller NL and Stock CC (1982) Lipid associated sialic acid test for the detection of human cancer. *Cancer Res* **42**: 5270–5275

Martin M, Rayo JI, Talavera JR, Munoz A and Cazino A (1990) Evaluation of serum tumor markers in the head and neck cancer. *J Nucl Med Allied Sci* **34**(suppl): 235–238

Maxim PE, Veltri RW, Sprinkle PM and Pusater RJ (1978) Soluble immune complexes in sera from head and neck cancer patients: a preliminary report. *Otolaryngology* **86**: 428–432

Moll R, Franke WW, Schiller DL, Geiger B and Krepler R (1982) The catalogue of human cytokeratin: pattern of expression in normal epithelia, tumors and cultured cells. *Cell* **31**: 11–24

Nakata T, Chung YS, Kato Y, Ogawa Y, Inui A, Maeda K, Sawada T and Sowa M (1996) Clinical significance of serum Cyfra 21-1 in gastric cancer. *Br J Cancer* **73**: 1529–1532

Niklinski J, Furman M, Chyczewska E, Chyczewski L, Rogowski F and Laudanski J (1995) Diagnostic and prognostic value of the new tumour marker Cyfra 21-1 in patients with squamous cell lung cancer. *Eur Respir J* **8**: 291–294

Niklinski J, Furman M, Burzykowski T, Chyczewski L, Laudanski J, Chyczewska E and Rapellino M (1996) Preoperative Cyfra 21-1 level as a prognostic indicator in resected primary squamous cell lung cancer. *Br J Cancer* **74**: 956–960

Ogawa T, Tsutusako Y, Kimura N, Nishioka S, Akagi H, Nishizaki K, Nishioka K and Rutka J (1999) Comparison of tumor markers in patients with squamous cell carcinoma of the head and neck. *Acta Otolaryngolo Suppl (Stockh)* **540**: 72–76

Osborn M and Weber K (1982) Intermediate filaments: cell-type-specific markers in differentiation and pathology. *Cell* **31**: 303–306

Plermo F, Carniato A, Fede A, Boccaletto F and Marchiori C (1990) Serum SCC antigen in head and neck squamous cell carcinoma. *Int J Biol Markers* **5**: 118–120

Pujol JL, Grenier J, Daures JP, Daver A, Pujol H and Michel FB (1993) Serum fragment of cytokeratin subunit 19 measured by Cyfra 21-1 immunoradiometric assay as a marker of lung cancer. *Cancer Res* **53**: 61–66

Pujol JL, Grenier J, Parrat E, Lehmann M, Lafontaine T, Quantin X and Michel FB (1996) Cytokeratin as serum markers in lung cancer: a comparison of Cyfra 21-1 and TPS. *Am J Respir Crit Care Med* **154**: 725–733

Rastel D, Ramaioli A, Cornillie F and Thirion B (1994) Cyfra 21-1, a sensitive and specific new tumour marker for squamous cell lung cancer. Report of the first European Multicenter Evaluation. *Eur J Cancer* **30A**: 601–606

*© 2000 Cancer Research Campaign*

Ropka ME, Goodwin WJ, Levine PA, Sasaki CT, Kirchner JC and Cantrell RW (1991) Effective head and neck tumor markers. *Arch Otolaryngol Head Neck Surg* **117**: 1011–1014

Shideler CE, Johns ME and Cantrell RW (1981) Erythrocyte polyamine determination in patients with head and neck cancer. *Arch Otolaryngol* **107**: 752–754

Silverman NA, Alexander JC and Chretien PB (1976) CEA levels in head and neck cancer. *Cancer* **37**: 2204–2211

Stieber P, Hasholzner U, Bodenmuller H, Nagel D, Sunder-Plassmann L, Dienemann H, Meier W and Fateh-Moghadam A (1993) Cyfra 21-1 a new marker in lung cancer. *Cancer* **7**: 707–713

Van der Gaast A, Schoenmakers CHH, Kok TC, Blijenberg BG, Cornillie F and Splinter TAW (1994) Evaluation of a new tumor marker in patients with non-small-cell lung cancer: Cyfra 21-1. *Br J Cancer* **69**: 525–528

Walther EK, Dahlmann N and Gorgulla HT (1990) Tumor markers in patients with head and neck carcinoma. *Laryngorhinootologie* **69**: 271–274

Wolf GT, Chretien PB and Elias EG (1979) Serum glycoproteins in head and neck squamous carcinoma. *Am J Surg* **138**: 489–500

Yen TC, Lin WY, Kao CH, Cheng KY and Wang SJ (1998) A study of a new tumor marker, CYFRA 21-1, in squamous cell carcinoma of the head and neck, and comparison with squamous cell carcinoma antigen. *Clin Otolaryngol* **23**: 82–86

Yoshimura Y, Oka M and Harada T (1990) SCC-antigen for detection of squamous cell and mucoepidermoid carcinoma after primary treatment: a preliminary report. *J Oral Maxillofac Surg* **48**: 1288–1292

*© 2000 Cancer Research Campaign*
